# Unraveling the Link Between Aortic Stenosis and Atherosclerosis: What Have We Learned?

**DOI:** 10.3390/medicina61081375

**Published:** 2025-07-30

**Authors:** Corina Cinezan, Camelia Bianca Rus, Ioana Tiberia Ilias

**Affiliations:** 1 Department of Medical Disciplines, Faculty of Medicine and Pharmacy, University of Oradea, 410073 Oradea, Romania; ioana.ilias@didactic.uoradea.ro; 2 Clinical County Emergency Hospital Bihor, 410169 Oradea, Romania; 3 Doctoral School of Biological and Biomedical Sciences, University of Oradea, 410087 Oradea, Romania

**Keywords:** valvular calcification, degenerative valve disease, risk factors, statins, PCSK 9, GLP1, siRNA inhibitors

## Abstract

*Background*: Aortic stenosis (AS) has long been considered a degenerative disease and is typically diagnosed in older men at an advanced stage. However, accumulating evidence has highlighted the similarities between AS and atherosclerosis, particularly regarding shared risk factors and overlapping pathophysiological mechanisms. This connection has led to a paradigm shift, suggesting that AS may be preventable in its early stages. *Methods*: This narrative review synthesizes the existing literature exploring the parallels between AS and atherosclerosis, focusing on common risk factors, pathogenic pathways, and evolving therapeutic strategies. Clinical trials and translational studies were examined to assess the effectiveness of atherosclerosis-based treatments for AS. *Results*: Multiple studies have confirmed the shared inflammatory, lipid-mediated, and calcific mechanisms of AS and atherosclerosis. Despite these similarities, therapeutic strategies effective in atherosclerosis, such as statin therapy, have not consistently shown benefits in AS. New medical approaches aim to delay aortic valve replacement and reduce the associated morbidity. The partially overlapping pathogenesis continues to guide future research. *Conclusions*: While AS and atherosclerosis share several pathogenic features, their clinical courses and treatment responses diverge. Understanding the limits and potential of their overlap may inform future preventive and therapeutic strategies. Earlier detection and targeted intervention in AS remain key goals, drawing on insights from cardiovascular disease management.

## 1. Introduction

Aortic stenosis represents an obstacle to left ventricular emptying, which can have multiple hemodynamic consequences. The obstruction of the ejection tract of the left ventricle is most commonly located at the level of the aortic valve; however, it can also be located below the valve (subvalvular) or above the valve (supravalvular) [1,2].

Degenerative aortic stenosis, previously called “senile”, is the most common cause of aortic stenosis in adults and the most common reason for aortic valve replacement [1]. 2% of people over 65 have been shown to have frank calcific aortic stenosis, while 29% have aortic valve sclerosis, defined as irregular thickening of the aortic valve leaflets, detected by echocardiography without significant obstruction of blood flow, in which the opening of the valve is not restricted and the maximum velocity of the blood at its level is less than or equal to 2.5 m/s. This is considered the mild or early phase of aortic stenosis [3,4]. The rate of progression from aortic sclerosis to aortic stenosis is 1.8 to 1.9% per year [2]. Aortic sclerosis has been found in 48% of people over the age of 84 years [5]. Aortic stenosis is a marker of increased cardiovascular risk^3^ and aortic sclerosis is associated with an increased risk of myocardial infarction [6]. An epidemiological association has been documented between calcific aortic stenosis and cardiovascular risk factors, suggesting that preventing or treating these factors may lessen the risk of developing or evolving aortic stenosis [2].

Calcific aortic valve disease was previously considered to be the result of years of normal mechanical stress on a normal aortic valve (wear and tear) [2].

Evidence has shown that degenerative aortic stenosis is an active disease [7,8], with active biological processes underlying the initiation and progression of calcific aortic valve disease [2] and having some common features with atherosclerosis. Immunological mechanisms are also involved in the etiology of degenerative aortic stenosis [9]. Chronic inflammation is a characteristic feature of aortic stenosis lesions [7,8,10].

Thus, the current tendency is to consider aortic stenosis, still called “degenerative,” not only as a condition associated with old age but also as the final part of an active pathological process [11]. This process involves proliferative and inflammatory changes in the valve, with lipid accumulation, increased tissue angiotensin-converting enzyme activity, infiltration with macrophages and lymphocytes, and finally, bone formation [1,3,9,10]. This process is initiated by lipid infiltration and oxidative stress, which attract and activate inflammatory cells and promote the elaboration of cytokines [12].

These processes may be similar to those observed in atherosclerosis.

The aim of this narrative review is to summarize the similarities between degenerative aortic stenosis and atherosclerosis and highlight potentially efficient therapies for aortic sclerosis.

## 2. Materials and Methods

This narrative review explores the shared pathophysiological mechanisms, risk factors, and clinical associations between aortic stenosis (AS) and atherosclerosis. A literature search was performed using the PubMed, Scopus, and Web of Science databases for articles published in English between 1984 and 2025. The following keywords and their combinations were used: “aortic stenosis,” “atherosclerosis,” “valvular calcification,” “vascular calcification,” “shared risk factors,” “inflammation,” “oxidative stress,” “lipoproteins,” and “molecular mechanisms.

Original research articles, narrative and systematic reviews, meta-analyses, and relevant clinical guidelines were included.

Studies focusing on aortic stenosis (AS) and/or atherosclerosis were also included, particularly those investigating shared epidemiological and cardiovascular risk factors (e.g., age, hypertension, dyslipidemia, diabetes mellitus, smoking), common molecular and pathophysiological mechanisms (e.g., inflammation, lipid accumulation, calcification, oxidative stress), biomarkers relevant to both diseases, therapeutic approaches targeting both conditions (e.g., statins, anti-inflammatory agents, PCSK9 inhibitors), human studies, and relevant animal models that provide mechanistic insights.

Studies focusing on pediatric populations, congenital valve abnormalities, or those unrelated to the intersection of AS and atherosclerosis were excluded.

After the initial screening of titles and abstracts, full-text reviews were conducted for potentially eligible articles. Additional studies were identified through the references of the selected papers. The selection process adhered to a narrative review framework, emphasizing thematic synthesis rather than quantitative meta-analysis. Priority was given to high-quality, peer-reviewed studies and recent evidence elucidating common pathophysiological pathways and their therapeutic implications.

The exclusion criteria were as follows: studies not published in English; articles unrelated to the pathophysiology or clinical interplay between aortic stenosis and atherosclerosis; case reports, conference abstracts, editorials, commentaries, and letters to the editor without original data or comprehensive analysis; studies focused exclusively on congenital valvular disease, rheumatic valve pathology, or pediatric populations; and duplicated publications or those lacking sufficient methodological or scientific rigor.

The final selection included 125 articles, which were analyzed and synthesized thematically across key domains: (1) epidemiological overlap, (2) shared risk factors, (3) inflammatory and molecular mechanisms, (4) diagnostic advances, and (5) therapeutic considerations.

## 3. Similarities Between Aortic Stenosis and Atherosclerosis

### 3.1. Risk Factors

The early stages of atherosclerosis, before the appearance of its characteristic lesions (atherosclerotic plaques) and clinical manifestations, are characterized by endothelial dysfunction [13]. The main risk factors are listed in Table 1.

Hypercholesterolemia is a risk factor for endothelial dysfunction and atherosclerosis. It favors the increase in the activity of caveolin—a protein in vascular endothelial cells—which inhibits nitric oxide synthase, with a decrease in nitric oxide generation and consequent endothelial dysfunction [14]. Caveolin has been identified in aortic valve endothelial cells [15]; thus, an increase in plasma cholesterol is associated with degenerative aortic stenosis [15,16].

Individuals with calcification of the mitral annulus and aortic valves have an increased incidence of atherosclerosis risk factors. Advanced age, female sex, arterial hypertension, diabetes, and hypercholesterolemia are associated with aortic valve calcification without stenosis, and age, arterial hypertension, and increased cholesterol levels are associated with aortic stenosis [17]. Valve changes occurred more frequently in females in this study, unlike in other studies [18], which showed that advanced age, male sex, increased cholesterol, LDL-cholesterol concentration, lipoprotein (a), smoking, hypertension, and diabetes are risk factors for degenerative aortic stenosis [19,20,21,22]. Body mass index and smoking have been shown to be independent predictors of significant progression of calcific aortic stenosis [23].

Lipoprotein(a) [Lp (a)] plays an important role in atherogenesis. One gene locus associated with lipoprotein(a) levels is strongly associated with calcific aortic valve stenosis in several racial groups [24,25]. High levels of lipoprotein(a) are associated with microcalcification and macrocalcification of the aortic valve [26,27,28], and together with oxidized phospholipid-apoB levels, may promote faster progression of aortic stenosis [29,30,31].

Aortic stenosis and sclerosis are associated with increased triglyceride levels and decreased HDL levels [21]. Kaltoft et al. [32] found that higher triglycerides and remnant cholesterol were associated with increased risk of aortic stenosis. An inflammatory mechanism may be involved, with elevated triglyceride-rich remnant lipoproteins being the drivers of aortic stenosis. Another study [33] found that low levels of LDL cholesterol probably prevent aortic aneurysm, aortic stenosis, and coronary artery disease, although they may increase the risk of thromboembolic events. In another study [34], an elevated apoA/apoA-1 ratio was associated with an increased incidence of aortic stenosis. Hypercholesterolemia experienced by rabbits fed a high-cholesterol diet produces changes in aortic valves [35,36] and apoptosis at that level [35].

Infection with Chlamydia pneumoniae, which has a proven role in the pathogenesis of atherosclerosis, is related to aortic sclerosis [37]; in association with increased values of lipoprotein (a) and C-reactive protein, it can influence and aggravate lesions from aortic valve sclerosis through the formation of circulating immune complexes [38]. Plasma leptin and tissue plasminogen inhibitors, considered risk factors for atherosclerosis [39], are associated with aortic sclerosis. The pathogenic role of Chlamydia pneumoniae in aortic sclerosis has been questioned in other studies [40].

Elevated levels of C-reactive protein have been identified in individuals with degenerative aortic stenosis [1].

In another study [41], none of the cardiovascular risk factors—male sex, hypercholesterolemia, smoking, diabetes, and family history of ischemic coronary disease— had a higher prevalence in patients with aortic stenosis. However, male sex, hypercholesterolemia, smoking, diabetes, and family history of ischemic heart disease were significantly associated with the presence of ischemic heart disease in patients with aortic stenosis. Thus, due to the association between aortic stenosis and ischemic heart disease, the risk factors correlate with coronary atherosclerosis in patients with aortic stenosis.

The progression of aortic stenosis consists of a decrease in the aortic valve area by an average of 0.1 cm^2^ per year and an increase in the aortic transvalvular pressure gradient by an average of 7 mm Hg per year. The factors associated with the progression of aortic stenosis are advanced age, male sex, dyslipidemia, smoking, arterial hypertension, diabetes, obesity, hypercalcemia, increased creatinine, and valvular calcification; some of these factors are found in the pathogenesis of atherosclerosis [21]. The evolution of calcium deposition in the aortic valves, assessed by electron emission computed tomography, was faster in patients with higher plasma LDL cholesterol levels than in those with lower LDL levels. The rate of progression of calcium deposition was not influenced by smoking, hypertension, diabetes, or age of the patients [42].

Hypertension, smoking, diabetes mellitus, and metabolic syndrome have been linked to the development of aortic stenosis, even if not all patients with risk factors for atherosclerosis develop calcific aortic stenosis, and not all patients with calcific aortic stenosis have risk factors for atherosclerosis. In patients with risk factors for atherosclerosis and aortic stenosis, the exact disease-promoting action is unclear [43,44].

Wal et al. [45] showed a link between aortic stenosis and diabetes mellitus, the latter negatively impacting the quality of life and longevity of patients with aortic stenosis.

In a study by Boudoulas et al. [46], 62.2% of patients with tricuspid aortic valve stenosis and 26.3% of patients with bicuspid aortic stenosis with aortic valve replacement required concomitant coronary artery by-pass surgery, with patients in the second category being younger than those in the first. In another study, the incidence of coronary artery disease was higher in these groups than in groups without aortic stenosis [47], suggesting that risk factors related to coronary atherosclerosis also play a role in the development of calcific stenosis. The anatomy of the aortic valve may also play a role in the development of aortic stenosis, regardless of atherosclerosis risk factors, as patients with bicuspid aortic valve develop aortic stenosis at a much younger age than those with tricuspid aortic valve [46].

Other factors may lead to aortic stenosis. Some studies have suggested that the size of the three leaflets in a normal aortic valve may vary slightly, and this, in addition to other genetic factors, may lead to aortic calcification and stenosis [48,49]. Thus, genetic predisposition also plays an important role in the development of calcific aortic valve disease [24,46,49].

**Table 1:** The main risk factors and parameters of atherosclerosis studied in aortic stenosis are described in the scientific literature.

Parameter	Effect	Reference
Age	Strongly and significantly associated with stenotic aortic valve calcification.	Boon A. et al., 1997 [17]
	Increasing prevalence with age	Chan KL et al., 2003 [20]
	Increasing prevalence with age	Novaro GM et al., 2003 [21]
	Increasing prevalence with age	Shavelle DM et al., 2005 [19]
Male gender	Associated with the progression of calcific aortic stenosis	Chan KL et al., 2003 [20]
	Associated with the progression of calcific aortic stenosis	Novaro GM et al., 2003 [21]
	Associated with the progression of calcific aortic stenosis	Shavelle DM et al., 2005 [19]
Body mass index	Independent predictor of significant progression of aortic stenosis	Ngo MV; 2001 [23]
moking	Smoking is an independent predictor of significant progression of aortic stenosis.	Ngo MV; 2001 [23]
	Risk factor associated with aortic stenosis	Chan KL et al., 2003 [20]
	Associated with the development of aortic stenosis, together with endothelial dysfunction	Novaro GM et al., 2003 [21]
	Associated with aortic stenosis	Sathyamurthy I et al., 2003 [43]
	Associated with calcific aortic valve disease	Freeman RV et al., 2005 [44]
	Associated with calcific aortic valve disease	Shavelle DM et al., 2005 [19]
Arterial Hypertension	Associated with stenotic aortic valve calcification	Boon A et al., 1997 [17]
	Associated with aortic stenosis	Chan KL et al., 2003 [20]
	Associated with the progression of aortic stenosis	Novaro GM et al., 2003 [21]
	Associated with aortic stenosis	Sathyamurthy I et al., 2003 [43]
	Associated with aortic stenosis	Freeman RV et al., 2005 [44]
	Associated with the progression of calcific aortic stenosis	Shavelle DM et al., 2005 [19]
Diabetes mellitus	May play a role in the etiology of aortic stenosis	Deutscher S et al., 1984 [22]
	Associated with aortic stenosis	Chan KL et al., 2003 [20]
	Linked to the appearance of aortic stenosis	Novaro GM et al., 2003 [21]
	Associated with aortic stenosis	Sathyamurthy I et al., 2003 [43]
	Associated with aortic stenosis	Freeman RV et al., 2005 [44]
	Associated with the progression of aortic valve disease	Shavelle DM et al., 2005 [19]
	Diabetes mellitus negatively impacts the quality of life and longevity of patients with aortic stenosis.	Wal P et al., 2023 [45]
Hypercholesterolemia	Risk factors associated with aortic stenosis	Boon et al., 1997 [17]
	Increased plasma level of cholesterol is associated with degenerative aortic stenosis.	Wilmshurst et al., 1997 [16]
	Increases the activity of caveolin and decreases nitric oxide generation	Feron O et al., 1999 [14]
	Apoptosis induced by hypercholesterolemia may be important in the mechanism of aortic stenosis.	Rajamannsn NM et al., 2001 [15]
	Produces changes in the aortic valves and apoptosis at that level	Rajamannan NM et al., 2002 [15]
	Development of aortic valve sclerosis in animals	Ivert T et al., 2003 [34]
LDL concentration	Role in the etiology of aortic stenosis	Deutscher S et al., 1984 [22]
	Role in aortic valve calcification	Pohle K et al., 2001 [42]
	Associated with aortic stenosis	Chan KL et al., 2003 [20]
	Prothrombotic effects and cytotoxicity for many cells	Novaro GM et al., 2003 [21]
	Associated with the progression of aortic valve stenosis	Shavelle DM et al., 2005 [19]
	Low LDL cholesterol levels may prevent aortic stenosis	Allara E et al., 2019 [33]
Decreased HDL	Related low HDL levels were correlated with aortic sclerosis	Novaro GM et al., 2003 [21]
Increased triglycerides	Aortic stenosis and sclerosis are associated with increased triglycerides.	Novaro GM et al., 2003 [21]
	An inflammatory mechanism; elevated triglyceride-rich remnant lipoproteins are the drivers of aortic stenosis	Kaltoft M et al., 2020 [32]
High levels of lipoprotein (a)	Lipoprotein (a) accumulates in aortic valves together with calcium deposition.	Thanassoulis G et al., 2013 [24]
	Risk factor for aortic valvular stenosis	Arsenault BJ et al., 2014 [25]
	Implicated in the hemodynamic evolution of aortic stenosis	Kamstrup PR et al., 2017 [31]
	Associated with calcification of the aortic valve	Despres AA et al., 2019 [26]
	Lowering lipoprotein (a) may slow aortic stenosis progression	Zheng GH et al., 2019 [28]
	Oxidized phospholipid apoB and elevated lipoprotein (a) are predictive of faster disease progression in patients with established aortic valvular stenosis.	Schnitzler JG et al., 2019 [29]
	The associated risk factor is not only due to cholesterol content but could also be due to the structure of lipoprotein (a), resembling plasminogen.	Langsted A et al., 2020 [30]
	Associated with calcification of the aortic valve	Kaiser Y et al., 2021 [27]
Elevated apoB/apoA-1 ratio	Associated with an increased incidence of calcific valve disease	Ivert T et al., 2021 [34]
Chlamydia Pneumoniae infection	Increases C-reactive protein levels, which aggravate lesions from aortic valve sclerosis mediated by circulating immune complexes	Glader CA et al., 2003 [38]
	Chlamydia Pneumoniae infection is related to the pathogenesis of aortic sclerosis.	Nystrom-Rosander C et al., 2003 [37]
	Role in the pathogenesis of aortic stenosis	Agmon Y et al., 2004 [40]
Hypercalcemia	Factor associated with the progression of stenosis	Novaro GM et al., 2003 [21]
Increased creatinine	Factor associated with the progression of stenosis	Novaro GM et al., 2003 [21]
Valvular calcification	Factor associated with progression of stenosis	Novaro GM et al., 2003 [21]
Anatomy of the aortic valve	Role in the development of calcific aortic valve disease	Boudoulas KD et al., 2018 [46]
Elevated levels of C-protein	Inflammation plays a role in progressive valve narrowing.	Dweck MR et al., 2012 [1]
Plasma leptin and tissue fibrinogen inhibitors	Risk factors associated with aortic stenosis	Ridker PM et al., 2005 [39]
Genetic predisposition	The size of the three aortic leaflets could be associated with aortic stenosis.	Boudoulas H et al., 2009 [49]
	Implicated in the etiology of aortic stenosis	Padang et al., 2012 [50]
	Implicated in the etiology of aortic stenosis	Thanassoulis G et al., 2013 [24]
	The size of the three aortic leaflets could be associated with aortic stenosis.	Boudoulas KD et al., 2013 [48]
	Implicated in the anatomy and in the calcification of the aortic valve	Boudoulas KD et al., 2018 [46]

### 3.2. Pathogenesis

The first description of the similarities between aortic valve disease and atherosclerotic coronary artery disease was made by Ashworth in 1946. Both diseases are characterized by calcific deposits, lipids, and inflammatory cells (macrophages). The first changes in the aortic valves have been described as an alteration of valve collagen and an accumulation of lipids, followed by calcification [51]. Osteopontin, a protein that modulates bone formation, has been identified in both aortic valves and atherosclerotic plaques [10,51].

T lymphocytes accumulate in the aortic valve, suggesting the involvement of an immunological mechanism [9], apolipoproteins B, (a), and E, which contribute to the formation of early lesions that also contain inflammatory cells [52]. Due to this inflammatory infiltrate rich in macrophages and lymphocytes, the expression of metalloproteinases [53,54] and metalloproteinase inhibitors [54], involved in the remodeling of the extracellular matrix, increases. These phenomena also occur during the formation and evolution of atheroma plaques [13].

Tenascin, a protein of the extracellular matrix, was identified in atherosclerotic plaques and in lesions from degenerative aortic stenosis [7,55]; osteopontin is present in both lesions. In advanced aortic stenosis, mature lamellar bone is produced and is involved in bone remodeling [56,57].

Angiotensin-converting enzyme and chymase are present in aortic valve lesions; they are expressed due to infiltration with macrophages and mast cells [58,59] and in association with LDL-cholesterol particles, which are considered to produce angiotensin-converting enzyme, a phenomenon also present in atherosclerotic plaques [60].

Similar to atherosclerotic plaques, early aortic valve lesions contain lipid deposits and macrophages infiltrated with T cells; disruption of the basal membrane of the valve leaf occursconstituting the initial insult; inflammatory cells enter the endothelium at this level, together with lipids, constituting a chronic inflammatory infiltrate. It extends from the endothelium to the central part of the valve. At the level of the aortic valve, mineralization is more prominent than in atherosclerotic plaques; dysmorphic calcification is present to a greater extent under the influence of osteopontin and genetic mechanisms, and the number of smooth muscle cells is lower [18,61].

A necropsy study [62] demonstrated that aortic valves harvested from adults of all ages, including young adults between 20 and 40 years of age, have lesions similar to atherosclerotic plaques, even in macroscopically normal valves.

Clinical events in atherosclerosis are determined by the instability of the atherosclerotic plaque. In aortic stenosis, they are due to mechanical changes in the valve itself. In the early stages of aortic stenosis, areas of inflammatory cells and lipids are spread among areas of normal valvular tissue, and the valve remains flexible. Later, these lesions converge, fibrosis and calcification of the valve occur, with the increase of its rigidity and the achievement of obstruction at the level of the ejection tract of the left ventricle [18].

Therefore, both degenerative aortic stenosis and atherosclerosis are derived from an active inflammatory process [63]. The presence of the first is associated with the presence of the second in different forms of manifestation. Aortic sclerosis has been shown to be associated with systemic endothelial dysfunction, which in turn is predictive of cardiovascular events [64].

Calcifications of the aortic valve are related to those of the coronary arteries and thoracic aorta [65,66]; thus, aortic sclerosis and stenosis are markers of coronary disease [67]. In patients with non-ischemic mitral regurgitation, coronary artery disease and aortic sclerosis are associated [68].

The presence of mitral annular calcifications correlates with a significant risk of coronary events [69] and is a predictor of cardiovascular morbidity and mortality and ischemic stroke. Calcifications in the heart are markers of cardiovascular risk [70].

Calcific valvular aortic stenosis is associated with mitral annulus calcifications [71,72], which, in turn, are predictive of cardiovascular disease, regardless of the presence or absence of cardiovascular risk factors [73].

The association between the presence of aortic valve calcification and atherosclerotic carotid disease has been demonstrated [74]. Calcification of the aortic valve and mitral annulus is a diagnostic parameter for systemic atherosclerosis and is related to the carotid intima-media index [75]. The degree of valvular sclerosis and this index are directly proportional [76]. The higher the intima-media index in patients with aortic stenosis, the greater the possibility of atherosclerosis in other territories. An intima-media index greater than 1.2 mm in patients with aortic stenosis is predictive of associated coronary artery disease [77]. Carotid intima-media thickness and carotid atherosclerotic plaques are independent risk factors for ischemic stroke. In conclusion, degenerative changes in the aortic valves increase the severity of atherosclerotic disease by increasing the incidence of atherosclerotic plaques [78]. They correlate with the intima-media thickness, which is a marker of significant coronary atherosclerosis. This is important in patients with aortic stenosis undergoing aortic valve replacement surgery, as coronary disease in these patients darkens the prognosis [79].

However, Miller et al. [80] found important differences in the mechanisms underlying oxidative stress in aortic stenosis and atherosclerosis. This mechanism is considered to be a key trigger for procalcific processes in these tissues. In atherosclerosis, mechanical forces lead to plaque formation; in simpler terms, areas of low blood flow, usually found at curves or vessel bifurcations, can experience altered shear stress, leading to inflammation and the initial stages of plaque formation [80]. In aortic valve calcification, the mechanism is different; increased pressure and stress during the cardiac cycle on aortic leaflets can lead to calcification, especially in the closed position of the valve. This mechanical stress can damage the leaflet tissue and initiate calcification [80].

Dweck et al. [81], using non-invasive imaging, compared aortic valve calcification and inflammation activity with that measured in atherosclerotic plaque and bone. They found important differences between the aortic valve, thoracic aortic atheroma, and skeletal bone, with active calcification appearing more pronounced in the stenotic aortic valve than in regions of aortic atheroma. In contrast, the opposite was true for inflammation. The authors suggested that active calcification has a fundamental role in the progression of aortic stenosis, independent of inflammation or calcific activity in the atheroma or skeletal bone, with inflammation having a lesser role in aortic stenosis. When calcification is already present in the valve, it appears to progress independently of external factors.

Rajamannan et al. [82] concluded that calcific aortic stenosis is an active process, from initial cellular changes in valve leaflets to the development of aortic stenosis, resulting in bone formation. The valve leaflets contain valve endothelial cells and valve interstitial cells, which may behave differently at different times due to triggering factors. The answers to these triggers are differentiation into a variety of other cell types, such as myofibroblasts and osteoblasts, and dysfunction. These factors include hemodynamic shear stress, solid tissue stresses, inflammatory cytokines, and growth factors [43,82].

Both atherosclerosis and aortic valve disease begin with an inflammatory stimulus, but their pathophysiological mechanisms are different. While atherosclerosis is impelled by inflammation and is susceptible to acute events, aortic valve calcification progresses slowly, determined by the stiffening of the leaflets over time. In aortic valve calcification, the process begins with inflammation and the accumulation of lipids, leading to the infiltration of inflammatory cells like mast cells, macrophages, and T-lymphocytes, elements that accentuate oxidative stress and microcalcification, the early stage of calcification, characterized by tiny calcium deposits within the valve tissue. In atherosclerosis, smooth muscle cells that contribute to the wall structure and tone in healthy arteries migrate to the site of wall injury, adopt various phenotypes, and can transform into osteoblast-like cells. In this way, small particles that act like calcium sites are released, promoting calcification [39,50,69].

In aortic stenosis, a large contributor to disease progression is calcification, with gradual obstruction of the valve, while in coronary atherosclerosis, the main mechanism is inflammation, and the events are acute, caused by plaque rupture and thrombosis [43].

Rajamannan [45,83] defined the term “osteocardiology” as the process in which atherosclerosis may differentiate to form “bone” in the heart, producing coronary and/or left heart calcification. In addition, elevated serum calcium levels are associated with an increased risk of atherosclerosis and aortic valve calcification. A higher serum calcium level is associated with a higher prevalence of aortic valve disease, as it is thought to promote atherogenesis through vascular calcification [43].

Lee SE et al. [84] found that the overall burden of coronary was associated with aortic valve calcification at baseline and that the progression of aortic valve calcification was associated with only the progression of calcified plaque volume, but not with the progression of non-calcified plaque volume. The authors suggested a likely disparity in the underlying mechanisms of calcification progression in aortic stenosis and coronary atherosclerosis.

The key role of parathyroid hormone (PTH) in both atherosclerosis and aortic valve calcification cannot be ignored. This hormone is crucial for the homeostasis of calcium and phosphorus; abnormal values of PTH, particularly hyperparathyroidism, can contribute to cardiovascular disease, especially vascular and valvular calcification. PTH affects vascular smooth cells, contributing to their transformation into osteoblast-like cells and leading to calcification [83,84].

### 3.3. Therapeutic Approach

The idea for medical treatment of aortic stenosis occurred some decades ago, targeting the common mechanism of atherosclerosis with the hope that aortic stenosis is a preventable disease. In view of the above, Table 2 presents the main medical interventions in aortic stenosis.

In atherosclerosis, statins change endothelial function, decrease the activity of macrophages, decrease inflammation, increase the activity of nitric oxide synthase, increase the levels of nitric oxide, improve endothelial dysfunction, and inhibit the proliferation of smooth muscle cells. These are the pleiotropic effects of statins, beyond the lipid-lowering effects. Given the etiopathogenetic similarities between degenerative aortic stenosis and atherosclerosis, statins may have similar effects in aortic stenosis [85].

Hypercholesterolemia is associated with degenerative aortic stenosis and is involved in its pathogenesis and progression. Trials with statins were conducted to determine whether they are indicated in patients with aortic stenosis, with or without dyslipidemia [86].

Statins slow the progression of coronary artery disease; therefore, they may reduce the progression of aortic stenosis [21]. They produce a smaller decrease in the aortic transvalvular peak gradient and a smaller decrease in valve area [21,87]. In aortic stenosis, they have anti-inflammatory effects, proven by a decrease in the level of vascular adhesion cells, as in atherosclerosis [88]. They could be beneficial in delaying the progression from aortic sclerosis to aortic stenosis [89].

The level of LDL-cholesterol is closely correlated with the rate of progression of aortic valve calcification, assessed by electron emission computed tomography; thus, lowering LDL can influence the progression of aortic stenosis [42,90]. This progression is not associated with cholesterol levels; however, statin treatment is associated with a slower progression of aortic stenosis [91].

Similar to atherosclerosis, statins reduce the secretion of metalloproteinases by activated valve macrophages and improve endothelial dysfunction by attenuating superoxide anion formation. Thus, they exert antiatherogenic effects at the level of the aortic valve [92].

Experimental hypercholesterolemia in rabbits resulted in valve mineralization with bone formation; atorvastatin inhibited this mineralization, with increased nitric oxide synthase expression and serum nitric oxide concentration [93].

Statins have been shown to decrease the progression of aortic stenosis in non-randomized, retrospective, observational studies. There is a need for intensive, long-term, randomized, controlled trials in patients with mild aortic stenosis [94].

C-reactive protein levels are increased in patients with degenerative aortic stenosis, both in the valve and serum, and both values decrease following statin treatment [95].

In the SEAS study (Simvastatin and Ezetimibe in Aortic Stenosis) [96], treatment with simvastatin and ezetimibe was not more effective than placebo in reducing aortic valve-related events and cardiovascular events, which were the primary targets of the study. Regarding secondary targets, the combination was significantly more effective than the placebo in reducing the risk of ischemic events, nonfatal myocardial infarction, coronary artery bypass graft surgery, percutaneous transluminal angioplasty, hospitalization for unstable angina, nonhemorrhagic stroke, and cardiovascular death. Experts believe that in patients with aortic stenosis who would not normally undergo lipid-lowering therapy and are at increased risk of atherosclerosis, simvastatin and ezetimibe therapy results in fewer complications of surgery and atherosclerosis.

This study highlights the need to examine patients with aortic stenosis from the perspective of their atherosclerotic risk. Therefore, patients with aortic stenosis could benefit from this therapy, even if they do not have dyslipidemia.

The prospective SALTIRE (Scottish Aortic Stenosis and Lipid Lowering Trial, Impact on Regression) study did not show the benefits of statins in patients with severe degenerative aortic stenosis. Atorvastatin does not delay or induce the regression of aortic stenosis [97]. Thus, statins should not be prescribed to patients with aortic stenosis unless they have another indication [97,98].

In the Rosuvastatin Affecting Aortic Valve Endothelium (RAAVE) study, asymptomatic patients with moderate aortic stenosis treated for an average of 73 weeks with rosuvastatin for elevated LDL-cholesterol had less progression of aortic stenosis than those who were not treated with statins [99].

ASTRONOMER [56] was a prospective study to determine the role of statins in patients with degenerative aortic valve disease—Aortic Stenosis Progression Observation: Measuring of Rosuvastatin. This showed that, despite favorable changes in lipid parameters and C-reactive protein levels, the progression of aortic stenosis was not influenced. Rosuvastatin did not slow progression of aortic stenosis, as measured by peak aortic gradient, in the subgroup analysis based on aortic stenosis severity, age, and severity of calcification of the valve.

The stage at which statin therapy is initiated may be important [43]. It is possible that statin therapy was initiated too late in the stage of the disease, when the inflammatory phase was exceeded, although patients with mild and moderate aortic stenosis were included in the study. Miller et al. [100] showed that reducing plasma lipid levels by genetic methods in hypercholesterolemic mice with early aortic valve disease halts the progression of aortic valve stenosis.

A meta-analysis by Zhao et al. [101] revealed that statin therapy does not prevent the progression of aortic stenosis. In atherosclerosis, statins attenuate the progression of coronary atherosclerosis and accelerate the calcification of atherosclerotic plaques, making the plaques more stable [102,103].

Angiotensin-converting enzyme accumulates in atherosclerotic plaques; it is produced by macrophages and vascular endothelial cells and is important in atherosclerotic pathology; the atherosclerotic plaque is the target of atherosclerosis therapy [104]. Experimentally, in monkeys, it was found that the regression of atherosclerotic lesions led to a decrease in angiotensin II [105]; thus, angiotensin II plays a role in atherosclerosis [106].

Angiotensin II, together with the receptor for angiotensin AT1 and the converting enzyme are expressed in arteries with atherosclerosis; angiotensin II is produced by angiotensin-converting enzyme in the atherosclerotic plaque and, in turn, they produce interleukin 6; thus, the renin—angiotensin system contributes to the inflammatory process in the arterial wall and to the development of acute coronary syndromes [107]. Renin-angiotensin-aldosterone system alterations are implicated in the pathophysiology of calcific aortic valve disease. It promotes increased LDL absorption in valvular lesions, inflammation, and a profibrotic state [58]. LDL particles release angiotensin-converting enzyme in aortic valve lesions, as well as in atheroma plaque [60].

ACE inhibitors, like statins, have proven useful in treating atherosclerosis. They have been shown to be beneficial in reducing overall cardiovascular mortality and morbidity in patients at high cardiovascular risk who do not have left ventricular dysfunction or congestive heart failure.

The use of angiotensin-converting enzyme inhibitors decreases atherogenesis by inhibiting the conversion of angiotensin I to angiotensin II, increasing nitric oxide levels, improving endothelial dysfunction, increasing bradykinin levels, and decreasing muscle proliferation. Angiotensin-converting enzyme inhibitors have not been shown to be beneficial in decreasing the rate of progression of aortic stenosis [85], even though they have been shown to reduce calcium accumulation at this level, as detected by electron emission computed tomography [58].

A retrospective analysis of patients with aortic stenosis [108] showed that the course of calcification of the aortic valve was slowed by medication that inhibits the renin-angiotensin-aldosterone system. However, there is no clear evidence that the use of this class of medications can improve the progression of aortic stenosis. In addition to their blood pressure effect, this medication might reduce fibrosis in stenotic aortic valves. The Angiotensin Receptor Blockers in Aortic Stenosis (ARBAS) study will investigate whether angiotensin-receptor-blockers can slow disease progression in the valve and delay fibrosis in the myocardium [109].

Novel LDL cholesterol-lowering protein convertase subtilisin/kexin type 9(PCSK9) inhibitors lower LDL cholesterol levels and those of lipoprotein(a). PCSK9 is a serine protease that promotes the degradation of the LDL receptor. Gain-of-function mutations in PCSK9 produce increased levels of LDL cholesterol and an increased risk of cardiovascular disease. Loss-of-function mutations are associated with lower levels of LDL-cholesterol and reduced cardiovascular risk [110]. PCSK 9 inhibitors reduce LDL and Lp (a) levels in the blood. The PCSK9 R46 L loss-of-function mutation has been associated with lower levels of LDL-cholesterol and a reduced risk of myocardial infarction [111]. Langsted et al. [110] showed that PCSK9 R46 L carriers have lower levels of Lp(a) and LDL cholesterol and also a reduced risk of myocardial infarction and of aortic stenosis, suggesting that PCSK9 inhibitors may have a role in patients with aortic stenosis.

Bergmark et al. [112] revealed in the FOURIER (Further Cardiovascular Outcomes Research with PCSK9 Inhibition in Subjects with Elevated Risk) trial that PCSK9 inhibition with evolocumab could decrease calcific aortic valve stenosis incidence in patients with cardiovascular disease.

Perrot et al. [113] showed that aortic stenosis was less prevalent in carriers of the PCSK9 R46 L variant and that PCSK9 is secreted by aortic valves. In addition, the authors revealed that PCSK9 inhibition in vitro might lower the calcification in aortic stenotic valves, suggesting that PCSK9 inhibition could be a future therapeutic option for these patients.

The role of Lp(a) in the pathogenesis of calcific aortic valve disease explains the limited role of statins in patients with this disease. It is well-known that statins and ezetimibe lower the levels of LDL-cholesterol, but not Lp(a) levels. Furthermore, statin use may increase Lp(a) levels. These findings, similar to those in atherosclerosis, highlight the potential role of PCSK9 inhibitors in reducing the progression of aortic stenosis.

Given the pathogenesis of aortic stenosis and the implicated inflammatory mechanism, other therapies effective against atherosclerosis may be promising. Xiao et al. [114] showed that the glucagon-like peptide 1 (GLP1) concentration in the calcific aortic valves was 39% less than in the control non-calcified valves and that this reduction in GLP1 was associated with calcific aortic valve disease. Zhou et al. [115] showed that liraglutide, a GLP1 agonist, attenuates aortic valve calcification in a high-cholesterol diet-induced experimental calcific aortic valve disease model in apolipoprotein E-deficient mice. Another GLP1 agonist, semaglutide, was superior to placebo in reducing the incidence of death from cardiovascular causes, nonfatal myocardial infarction, or nonfatal stroke in patients with preexisting cardiovascular disease and overweight or obesity [116]. So GLP1 agonists may be an important medication for patients with atherosclerosis and those with calcific stenosis.

Observational and experimental studies suggest that non-vitamin K oral anticoagulants may reduce the progression of valvular aortic stenosis, influencing fibro-calcific remodeling and inflammation [117]. Di Lullo et al. [118] reported that rivaroxaban, a factor Xa inhibitor, reduced valvular calcium deposits, aortic stenosis progression, and serum CRP levels compared with warfarin in patients with chronic kidney disease. Dabigatran, a direct thrombin inhibitor, and rivaroxaban and apixaban, which are also factor Xa inhibitors, have not only anticoagulant effects but also anti-inflammatory effects when studied in animals and in vitro [118,119,120,121,122,123,124,125]. In atherosclerosis, studies in mice revealed that dabigatran and rivaroxaban reduced lipid deposition, collagen content, macrophage accumulation, and the expression of inflammatory mediators [120,121,122,123,124,125].

With the intention of emphasizing the best therapies for atherosclerosis and aortic valve disease, despite the fact that both diseases share some common risk factors, the pharmacological therapy has been, in most cases, unsuccessful in treating calcific aortic valve stenosis, so the aortic valve replacement remains the primary treatment for severe cases. Although inflammation plays a role in aortic valve calcification, targeting this pathophysiological process has not been efficient. Other pharmacological approaches, such as statins, ACE inhibitors, PCSK9 inhibitors, or DPP-4, have not demonstrated many benefits in aortic stenosis outcomes, as mentioned previously. Although further research involving all these pharmacological classes is ongoing, the complexity of this disease may be the most important factor in the failure of many medications mentioned above. Currently, no approved drug therapy is used to stop and prevent aortic valve calcification [117,118,119,120,121,122,123,124,125].

**Table 2:** The most important medical intervention for aortic stenosis.

Medication/Study	Effect on Aortic Stenosis	Reference
ASTRONOMER—statins	Rosuvastatin did not slow the progression of aortic stenosis	Mohler et al., 2001 [51]
A retrospective analysis—ACE inhibitors	Calcification of the aortic valve was slowed by ACE inhibitors	Rosenhek R et al., 2004 [108]
SALTIRE study—statins	Atorvastatin does not delay or induce regression of aortic stenosis.	Cowell SJ et al., 2005 [97]
RAAVE study— rosuvastatin	Statins slowed the progression of aortic stenosis.	Moura LM et al., 2007 [99]
SEAS study—Simvastatin and Ezetimibe in Aortic Stenosis	Treatment with simvastatin and ezetimibe was not more effective than placebo in reducing aortic valve-related events and cardiovascular events.	Rossebo AB et al., 2008 [96]
Statins	Reducing plasma lipid levels by genetic methods in hypercholesterolemic mice with early aortic valve disease halts the progression of aortic valve stenosis.	Miller JD et al., 2009 [100]
PCSK 9 inhibitors	May have a role in patients with aortic stenosis	Langsted et al., 2016 [110]
Meta analysis—statins	Statins accelerated the calcification of atherosclerotic plaque, becoming more stable	Zhao Y et al., 2016 [101]
Rivaroxaban	Rivaroxaban reduced valvular calcium deposits, aortic stenosis, and CRP levels.	Di Lullo et al., 2019 [118]
FOURIER study—evolocumab	Inhibition of PCSK9 could decrease. calcific aortic valve stenosis incidence	Bergmark et al., 2020 [112]
PCSK9 inhibitors	The PCSK9 inhibition might lower the calcification in aortic stenosis valves.	Perrot et al., 2020 [113]
Glucagon—like peptide 1	The glucagon-like peptide 1 -GLP 1 concentration in the calcific aortic valves is less than in the normal aortic valves.	Xiao et al., 2021 [114]
Liraglutide	Liraglutide lowers aortic valve calcification.	Zhou et al., 2023 [115]

## 4. Conclusions and Future Directions

Aortic stenosis and atherosclerosis share some similarities, but they are not the same diseases. They have common risk factors, or perhaps, given that aortic disease is present, especially in older people with atherosclerosis, these risk factors are coincidental. In addition, the two entities share some common mechanisms.

The cost of treating calcific aortic valve disease is rising, and no medication is effective in delaying the need for surgery. Statins, which save lives in atherosclerosis, do not interact with disease progression despite some encouraging results. In aortic sclerosis and stenosis, inflammation is primordial, so it is possible that only certain time periods of disease development are susceptible to modulation targeting this mechanism, including lipid-lowering therapy. However, LDL-cholesterol accumulation may not be the most important or only point of interest in aortic stenosis pathogenesis. The role of Lp(a) in valvular inflammation is therefore crucial. Targeting Lp(a) is promising; therefore, there may be room for other medications to slow the progression of aortic valvular disease, like PCSK9 inhibitors.

Atherosclerosis and aortic stenosis almost always coexist in clinical practice; therefore, the three groups of drugs- statins, inhibitors of the renin-angiotensin axis, and PCSK9 inhibitors–deserve their place and indication in patients with aortic stenosis. Perhaps, non-vitamin K novel anticoagulants can also do this.

A new class of drugs that lowers LDL-cholesterol and Lp(a) levels has shown promise. This is a proprotein convertase subtilisin/kexin type 9(PCSK9) and small interfering RNA (siRNA)-based drug that inhibits intracellular PCSK9 synthesis, representing a new strategy for managing lipid disorders and reducing cardiovascular risk. Inclisiran is a long-acting, synthetic siRNA that targets the hepatic production of PCSK9 and reduces LDL-cholesterol levels by approximately 50% compared with placebo [126]. In the future, aortic stenosis may be the target for this group of medications.

Given that atherosclerosis and aortic stenosis share some similarities, we must consider all successful interventions for cardiovascular diseases based on atherosclerosis in patients with calcific aortic stenosis.

Further research is needed to study the genetic implications of valvular pathology and its molecular pathways. A better understanding of aortic sclerosis and stenosis initiation and progression will lead to a better therapeutic strategy to optimize current disease management and prevent its development.
